# Validation and Application of Skin RT-QuIC to Patients in China with Probable CJD

**DOI:** 10.3390/pathogens10121642

**Published:** 2021-12-19

**Authors:** Kang Xiao, Xuehua Yang, Wei Zhou, Cao Chen, Qi Shi, Xiaoping Dong

**Affiliations:** 1 State Key Laboratory for Infectious Disease Prevention and Control, National Institute for Viral Disease Control and Prevention, Chang-Bai Rd 155, Beijing 102206, China; xiaokang@ivdc.chinacdc.cn (K.X.); yangxuehua@ipbcams.ac.cn (X.Y.); zhouwei@ivdc.chinacdc.cn (W.Z.); chencao@ivdc.chinacdc.cn (C.C.); 2 Collaborative Innovation Center for Diagnosis and Treatment of Infectious Diseases, Zhejiang University, Hangzhou 310007, China; 3 Center for Biosafety Mega-Science, Chinese Academy of Sciences, Wuhan 430071, China; 4 China Academy of Chinese Medical Sciences, Dongzhimeinei, South Rd 16, Beijing 100700, China; 5 Center for Global Public Health, Chinese Center for Disease Control and Prevention, Chang-Bai Rd 155, Beijing 102206, China

**Keywords:** prion disease, sCJD, RT-QuIC, skin, diagnosis, biomarker, seeding activity

## Abstract

The definite diagnosis of human sporadic Creutzfeldt–Jakob disease (sCJD) largely depends on postmortem neuropathology and PrP^Sc^ detection in the brain. The development of real-time quaking-induced conversion (RT-QuIC) of cerebrospinal fluid (CSF) samples makes it possible for premortem diagnosis for sCJD. To test the diagnostic potential of RT-QuIC of skin specimens for probable sCJD, we collected the paired skin and CSF samples from 51 recruited living patients referred to the Chinese CJD surveillance center, including 34 probable sCJD, 14 non-CJD, and 3 genetic prion disease (gPrD). The samples were subjected to RT-QuIC assays using recombinant hamster PrP protein rHaPrP90-231 as the substrate. Using skin RT-QuIC assay, 91.2% (31/34) probable sCJD patients, and 1 T188K genetic CJD (gCJD) cases showed positive prion-seeding activity, while 85.7% (12/14) non-CJD patients were negative. CSF RT-QuIC positive seeding activity was only observed in 14 probable sCJD patients. Analysis of the reactivity of 38 positive skin RT-QuIC tests revealed that the positive rates in the preparations of 10^−2^, 10^−3^ and 10^−4^ diluted skin samples were 88.6% (39/44), 63.6% (28/44), and 25.0% (11/44), respectively. Eleven probable sCJD patients donated two skin specimens collected at different sites simultaneously. Although 95.5% (21/22) skin RT-QuIC elicited positive reaction, the reactivity varied. Our preliminary data indicate high sensitivity and specificity of skin RT-QuIC in prion detection for Chinese probable sCJD and highlight that skin prion-seeding activity is a reliable biomarker for premortem diagnosis of human prion disease.

## 1. Introduction

Human prion disease (PrD) is a group of transmissible neurodegenerative diseases, consisting of sporadic form, e.g., sporadic Creutzfeldt–Jakob disease (sCJD), genetic or familial form, e.g., genetic CJD (gCJD), fatal familial insomnia (FFI), Gerstmann–Sträussler–Scheinker disease (GSS), and acquired form, e.g., iatrogenic CJD (iCJD) and variant CJD (vCJD) [1,2,3]. sCJD is the most common form of human PrD with the incidence of one to two patients per million per year. The definite diagnosis of human PrD, particularly for sCJD, is still largely dependent on postmortem brain tissues showing special neuropathological changes, i.e., spongiform degeneration, and/or PrP^Sc^ deposit [2], although some biomarkers in cerebrospinal fluid (CSF) have revealed significances in diagnosis of sCJD, such as 14-3-3 and tau [4].

In the past decade, the diagnostic predicament for CJD has been greatly improved due to the development of the real-time quaking-induced conversion (RT-QuIC) assay [5]. The implementation of RT-QuIC in the detection of prions in CSF samples has been verified to be of the meaningful diagnostic value for sCJD clinically [5,6]. Some countries, such as the USA and UK, have even listed the CSF RT-QuIC test in the national diagnostic standard or criteria for sCJD. However, the sensitivities of CSF RT-QuIC assays in sCJD diagnosis may vary among the different laboratories, ranging from 60% to almost 100% [5,7,8,9,10]. On the other hand, collecting CSF samples through spinal taps is an invasive process in clinics, and some patients fail to provide CSF due to contraindications or refusal of lumbar puncture. The feasibility of usage of RT-QuIC in other easily obtained specimens is mostly desired.

Recently, the detection of prions in skin specimens with a RT-QuIC test has been reported by Zou’s team. They have found that prions could be detected from the autopsy skin tissues of sCJD and vCJD patients [11]. Based on animal experiments, they have also proved that prions could be detected in skin tissues even before the onset of clinical symptoms [12]. These results highlight a good applied prospect of skin RT-QuIC in the premortem diagnosis of human PrDs. In this study, we obtained the paired skin and CSF samples from 51 recruited living patients referred to the Chinese CJD surveillance center, including 34 probable sCJD, 3 gPrD and 14 non-CJD. Positive reaction in a skin RT-QuIC assay was observed in 34 sCJD cases. Meanwhile, only 14 out of 34 probable sCJD were positive in CSF RT-QuIC tests. Our results indicated that a skin specimen was ideal for a RT-QuIC test in Chinese patients.

## 2. Results

Our CSF RT-QuIC assay was blindly evaluated previously with a sensitivity of 96.7% and a specificity of 100% by CSF samples of neuropathologically confirmed 30 definite sCJD and 30 non-CJD provided kindly by the NPDPSC [13]. In this study, the skin samples of the coded 10 definite sCJD patients and 5 non-CJD controls from NPDPSC with a dilution from 10^−2^ to 10^−4^ were examined blindly by our RT-QuIC assay. All the samples were from the site of the lower back. The main demographic information of these 15 subjects is summarized in Table 1. RT-QuIC assays revealed the positive reactions in all definite sCJD samples and negative reactions in all non-CJD samples (Table 1), indicating 100% sensitivity and specificity of our RT-QuIC assay for prion detection in the skin samples of sCJD and controls.

The paired skin and CSF samples from 51 patients with different clinical diagnoses were enrolled for RT-QuIC assays in this study. The main demographic, clinical examinations and laboratory tests of the 51 patients were summarized in Table 2. The median of the onset age of 34 probable sCJD cases was 61.3 years (y) old (ranging from 28 to 84 y), while that of 14 non-CJD cases was 62.8 y (43–83 y). The majority of the patients were MM homozygous at codon 129 and EE homozygous at codon 219 of the prion protein gene (*PRNP*). Periodic sharp wave complexes (PSWC) on EEG were recorded in 8 of 31 sCJD cases and the T188K gCJD case, but not in 13 non-CJD cases who undertook the examination of EEG. sCJD associated abnormalities (high signal in caudate/putamen and/or symmetrical or dissymmetrical cortical ribbon syndrome on diffusion-weighted imaging (DWI)) were noticed in 24 of 33 sCJD patients, T188K and G114V gCJD cases, as well as 8 of 12 non-CJD cases, particularly the cortical ribbon syndrome. CSF 14-3-3 protein was positive in 16 of 30 sCJD cases, the G114V gCJD case, but also in 5 of 14 non-CJD patients (Table 2).

**Figure 1:**
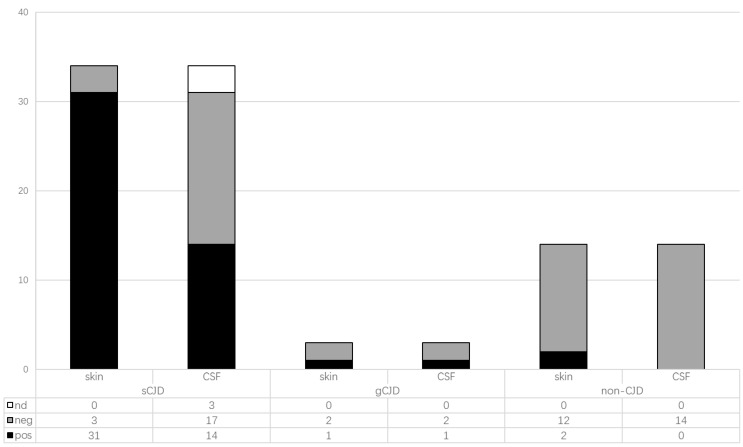
The positive and negative numbers in the skin and CSF RT-QuIC assays among the patients with different diagnosis.

Aliquot of 15 µL CSF samples from each patient were subjected to RT-QuIC tests. Only 14 sCJD cases and the T188K gCJD case revealed positive reactions, while the rest of the CSF samples tested were negative (Table 2, Figure 1). Differently diluted skin homogenates from 51 patients were also employed in RT-QuIC assays. As shown in Table 2 and Figure 1, most of the sCJD cases were positive (31/34), whereas 12 of 14 non-CJD cases were negative. Additionally, the T188K gCJD cases were also positive in RT-QuIC tests, while the G114V gCJD and D178N FFI cases were negative. Those data highlight that RT-QuIC reactivity or sensitivity of skin samples of sCJD patients is higher than that of CSF samples.

Furthermore, the RT-QuIC reactivity of each positive skin sample was analyzed based on the dilution of the skin homogenate and the reactivity in every testing well. As shown in Table 3, the general positive rates of the tested skin samples revealed a notable dose-dependent pattern, showing 88.6% (39/44) positive rate in the reaction of 10^−2^ dilution, 63.6% (28/44) in that of 10^−3^ and 25.0% (11/44) in that of 10^−4^ dilution. The RT-QuIC positive reactivity of the most tested samples reduced along with the increase of dilution, with four exceptions. Two showed positive reaction in the preparation of 10^−3^, but negative in those of 10^−2^ and 10^−4^, while the other two were positive in the reaction of 10^−4^, but negative in that of 10^−2^ and 10^−3^. It seems that the skin samples of human prion diseases possess the similar dose-dependent RT-QuIC reactive profile as the brain samples.

Fourteen patients (sCJD Cases 24 to 34, as well as non-CJD Cases 12 to 14) donated two skin samples simultaneously, collected from different sites including posterior neck, lateral malleolus, medial forearm, behind ear and inner thigh. Most of these samples (12/14) showed consistent results except sCJD Case 34 and non-CJD Case 14. sCJD Case 34 was positive in the sample from lateral malleolus but negative in the posterior neck. Non-CJD Case 14 was positive in the left medial upper arm but not the right arm. The reactivities of these 14 patients were also slightly different. The samples of posterior neck in the sCJD Case 24 and 33 were positive in the reactions of all dilutions (10^−2^ to 10^−4^), while the samples of lateral malleolus from the same patients elicited positive RT-QuIC reactions only in the dilutions of 10^−2^ and 10^−3^ (Table 3). By contrast, sCJD Case 34 was positive in the sample of lateral malleolus but negative in the sample of the posterior neck. 

To evaluate the potential influence factors for the reactivity in skin RT-QuIC test, 51 tested patients were grouped based on the reactivities of the skin RT-QuIC (34 positive and 17 negative) and the associations with some main data of clinical history, clinical examinations and laboratory tests were analyzed. As shown in Table 4, despite slight differences between the skin RT-QuIC positive and negative groups, no statistical significance was observed in age of disease onset, gender, and polymorphisms at residues s 129 and 219 of PrP. PSWC on EEG seemed to be a positive, related factor to the positive reaction in skin RT-QuIC, as 8 out of 23 patients with positive in skin RT-QuIC reported PSWC on EEG, while only 1 out of 15 cases who were negative in skin RT-QuIC revealed PSWC on EEG, however, without statistical significance (*p* = 0.06). Special abnormalities on MRI were recorded in 25 out of 33 patients (one case not performed) showing the positive skin RT-QuIC, and also observed in 9 out 15 cases (two cases not performed) in the negative group. CSF 14-3-3 positive was detected in 15 out of 32 cases (two case not performed) in the positive group and 7 out 15 cases in the negative one. All 15 patients with CSF RT-QuIC positive were also skin RT-QuIC positive. 

## 3. Discussion

CSF RT-QuIC assay is becoming an ideal method for the premortem diagnosis of sCJD and prion research based on its reliable specificity and high sensitivity [5,8]. Besides RT-QuIC, another similar technique, endpoint quaking-induced conversion (EP-QuIC), was also adopted in some countries such as Canada. Both assays exploited the ability of PrP^Sc^ to induce the conversion of PrP^C^ into a misfolded form. However, RT-QuIC has a better specificity and a slightly lower sensitivity [14]. Considering that sCJD is a non-communicable disease with nearly 100% mortality, specificity should be put in the first place. In this study, we tested the feasibility of the skin RT-QuIC assay in the diagnosis for probable sCJD together with the paired CSF samples. We found that 91.1% (31/34) of probable sCJD cases were positive in skin RT-QuIC assays using recombinant hamster PrP90-231 as the substrate, whereas 14.3% (2/14) cases who were considered as non-CJD at the time of sampling were positive. It should be noted that these two cases showing positive reactions in the group of non-CJD were lost to follow up. However, at the times of sampling, the clinical manifestations did not meet the diagnostic criteria. Moreover, only 45.2% (14/31) paired CSF samples from those probable sCJD cases showed positive in CSF RT-QuIC. It highlights higher sensitivity of skin RT-QuIC for prion detection in the Chinese probable sCJD cases than that of CSF RT-QuIC. Although the exact reasons why our CSF RT-QuIC assay reveals a lower sensitivity compared to the previous reports by others remain unclear [10], the following possibilities need to be excluded in the future. First, it could be due to the high prevalence of sCJD with 129 MM that was found in all cases except for one and that has been shown to have a lower sensitivity compared to sCJD with 129VV. Second, it could result from some unknown inhibitors in CSF, which shows a race-related tendency, as under the same RT-QuIC experimental condition a very high positive rate (96.7%) was detected in the CSF samples from US Caucasian patients. Certainly, we cannot exclude the possibility of relatively low sensitivity of the RT-QuIC in our experimental condition, which is able to detect PrP^Sc^ in CSFs of definite sCJD patients but shows low sensitivity in CSFs from probable sCJD cases.

Another point that needs to be emphasized was that since the specific MM type was not clear in Chinese patients, and there was no MM2 type in the definite samples from NPDPSC, we cannot draw a conclusion for skin RT-QuIC of MM2 samples yet.

Our data here revealed a dose-dependent manner of skin prion-seeding activity by the RT-QuIC assay. The highest positive rate was observed in the reactions of the lowest dilution (10^−2^ dilution). On the contrary, our study and many other studies have demonstrated that the brain tissues of sCJD patients can induce a positive reaction in RT-QuIC at very high dilution (10^−8^ dilution) [15,16]. Obviously, the reactivity of RT-QuIC depends on the amount of tissue PrP^Sc^. Usually, it is almost impossible to detect PrP^Sc^ in the skin specimen of CJD patients with routine techniques, such as protease- or guanidine-treated Western blot and immunohistochemistry, suggesting very low concentration of PrP^Sc^ in skin specimens. It is worth noting that a few skin samples in this study exhibited positive reactions at relatively higher dilutions (10^−3^ and 10^−4^) but failed to elicit positive reactions at low dilution (10^−2^). The exact reason is unknown at present. Our previous CSF RT-QuIC assay of human PrDs also revealed a similar phenomenon that excessive amounts of CSF from some patients had a low or no reactivity of prion-seeding activity. It is possible that some unknown inhibitors in human tissues may affect the RT-QuIC reactivity. Relatively shorter lag times and higher fluorescent intensities were observed in the low dilutions (10^−2^ and 10^−3^) of the skin samples generally (data not shown), but those features varied largely among the tested skin samples. Nevertheless, based on our preliminary data we suggest that it is better to perform skin RT-QuIC assays within a certain range of dilutions, such as 10^−2^ and 10^−3^ of tissue homogenate, for the diagnosis of human PrDs. 

We have also noticed that the skin samples showing positive reactions in skin RT-QuIC assays were collected from different body regions, including neck, upper arm, inner thigh, lateral malleolus, which implies a wide distribution of prion agents in skin tissues of sCJD patients during diseases. Zou and his colleagues have described that the skin specimens of sCJD patients around the head and ear seem to show the highest sensitivity in RT-QuIC test [11]. However, such a phenomenon is uncertain in the current study possibly due to limited tested numbers. We recommend taking multiple parts of the skin samples if it is possible to increase the identifying opportunity of prion agents with RT-QuIC.

The skin samples from three different gPrD cases were also included in this study. Only the case of T188K gCJD was positive in the skin RT-QuIC test. T188K gCJD is the second most frequently observed gCJD type in Chinese people [17]. More importantly, T188K gCJD is obviously predominant among Chinese patients, but rarely described in other countries, even in Japan and Korea [18]. Our previous study has revealed that 52% (13/25) T188K gCJD cases are positive in CSF RT-QuIC [19]. Coincidentally, the T188K gCJD case in this study was also positive in CSF RT-QuIC. D178N FFI is the most frequently detected gPrD in the Chinese [17]. The clinical phenotype and neurological abnormality of D178N FFI are markedly different from GSS and other gCJDs [20]. Our study has verified a remarkably low positive rate and weak reactivity in CSF RT-QuIC of FFI patients compared with the cases of E200K and T188K gCJD [19]. It is not surprising that the FFI case here was negative in skin RT-QuIC. G114V gCJD is rare subtype among the Chinese and only four cases have been identified in our surveillance system since 2006 [17]. Besides the case presented here, another G114V gCJD Chinese case showed negative in CSF RT-QuIC as well (data not shown). Due to the diversity of human gPrDs, the diagnostic significance of skin RT-QuIC needs further analysis.

Reviewing some clinical features of the tested patients with sCJD did not show any significant correlation with the results of skin RT-QuIC. Some sCJD associated clinical examination and laboratory tests seemed to be statistically correlated with the reactivity in skin RT-QuIC, such as PSWC on EEG, implying its accuracy for the diagnosis of sCJD. Therefore, it is most likely that those skin RT-QuIC associated factors probably reflect the close disease-related phenomenon. Although the skin RT-QuIC assay is still at the starting stage, our preliminary data revealed its high sensitivity in probable sCJD patients and reliable specificity in non-CJD cases, which provides a proof of concept that skin RT-QuIC is a suitable tool for premortem diagnosis of sCJD. 

## 4. Materials and Methods

### 4.1. Samples

The paired skin and CSF samples from 51 patients with probable sCJD, gPrD or non-CJD were obtained from the tissue bank in the Center of Chinese CJD Surveillance System, including 34 sCJD, 1 T188K gCJD, 1 G114V gCJD, 1 D178N FFI, and 14 cases who were considered as non-CJD cases at the time of sampling. Among them, 14 patients provided two skin samples collected from different sites (Table 2). The demographic information of the patients, the clinical data, MRI and EEG data, the results of the Western blot for CSF 14-3-3 and *PRNP* sequencing were collected from the database of the Center of Chinese CJD Surveillance System. 

In addition, 10% skin homogenates of neuropathologically-confirmed 10 sCJD patients and 5 non-CJD controls from the National Prion Disease Pathology Surveillance Center (NPDPSC), Case Western Reserve University School of Medicine, Cleveland, Ohio, USA, were also enrolled in this study for validation of our skin RT-QuIC assay. All five non-CJD subjects were determined by neuropathological assays on postmortem brains, which excluded the possibilities of sCJD or other human prion diseases but did not have a diagnosis for other neurological diseases. 

All enrolled CSF samples were obtained by lumbar puncture without visible blood. Routine CSF biochemistry assays of those specimens, including cell count, glucose and total protein were all in the normal ranges. To set up our CSF RT-QuIC assay, CSF samples from neuropathologically-confirmed CJD (*n* = 30) and non-CJD (*n* = 30) provided by NPDPSC were used. 

### 4.2. Preparation of Homogenates of Skin

The sites for skin biopsies in this study included the skins behind the ears, inside the arms, inside the thighs, lower back, and/or abdomen. After disinfection with 75% alcohol, the patient received local anesthesia with subcutaneous injection of 1–2% lidocaine hydrochloride. A small piece of skin with a size of about 0.2 × 0.3 cm^2^ was taken with a disposable skin biopsy punch (Acupunch, Acuderm Inc., Fort Lauderdale, FL, USA), according to the manufacturer’s instruction. Usually, the biopsy skin specimen covers epidermis, dermis, and adipose tissues. We prepared 2% (*w*/*v*) of skin homogenate in lysis buffer (900 µL of TBS containing 2 mM CaCl2 and 0.25% (*w*/*v*) collagenase A) according to a previously described protocol [11,12]. The homogenates were then stored at −80 °C for further used.

### 4.3. Real-Time Quaking-Induced Conversion RT-QuIC Assays

The detail procedure of the RT-QuIC assay was described previously [19]. Briefly, RT-QuIC reaction contained 10 µg of rHaPrP90-231, 1X PBS, 170 mM NaCl, 1 mM EDTA, 0.01 mM ThT, 0.002% SDS, together with 15 µL CSF samples or 2 µL 10^−2^ to 10^−4^ diluted skin homogenates in a final volume of 100 µL. Each sample was assayed in triplicate or quadruplicate. The assay was conducted in a black 96-well, optical-bottomed plate (Nunc, 265301) on a BMG FLUOstar plate reader (BMG LABTECH). The main working conditions were fixed as follow: temperature, 55 °C; vibration speed, 700 rpm; vibration/incubation time, 60/60 s; total reaction time, 60 h. ThT fluorescence (excitation wavelength, 450 nm; emission wavelength, 480nm) for each reaction was automatically measured every 45 min and expressed as relative fluorescence units (rfu). The cutoff value was set as the mean value of the negative controls plus 10 times the standard deviation. A sample was considered as positive when ≥2 wells revealed positive reaction curves. The positive control was 10^−5^ dilution of the brain homogenate of the scrapie agent 263K-infected hamster, while the negative control was 10^−5^ dilution of the brain homogenate of normal hamster.

### 4.4. Ethics Approval

Usage of the CSF and skin samples and relevant clinical information of the patients with different diseases in the Center of Chinese CJD Surveillance System has been approved and monitored by the Ethics Committee of the National Institute for Viral Disease Control and Prevention, China CDC, under the protocol of 2009ZX10004-101 and by the institutional review boards (IRB) of the University Hospital Cleveland Medical Center Written informed consent was obtained from family members for skin autopsy through the NPDPSC. 

### 4.5. Statistical Analysis

Statistical analysis was performed using SPSS 17.0 statistical package (Armonk, NY, USA). Levene’s test and Student’s *t*-test were used as appropriate. *p* value less than 0.05 was considered to be statistically significant.

## Figures and Tables

**Table 1:** The demographic information of 15 cases from the US Creutzfeldt–Jakob disease (CJD) surveillance center.

Case No.	Onset Age	Gender ^1^	Diagnosis	Skin RT-QuIC
Case 1	68	F	Non-CJD	−
Case 2	78	M	Non-CJD	−
Case 3	84	F	Non-CJD	−
Case 4	65	M	Non-CJD	−
Case 5	58	M	Non-CJD	−
Case 6	68	M	sCJD ^2^ MV1-2 ^3^	+
Case 7	59	F	sCJD MM1	+
Case 8	65	F	sCJD MM1-2	+
Case 9	67	M	sCJD MM1	+
Case 10	79	M	sCJD MV2	+
Case 11	60	F	sCJD MV1-2	+
Case 12	57	F	sCJD MM1	+
Case 13	71	F	sCJD VV2	+
Case 14	78	F	sCJD MM1	+
Case 15	68	F	sCJD MM1	+

^1^ Female (F); Male (M); ^2^ sporadic Creutzfeldt-Jakob disease; ^3^ methionine (M); valine (V).

**Table 2:** The main clinical and laboratory information of the patients with different diseases from Chinese CJD surveillance system.

Diagnosis	Onset Age	Gender	Codon 129 ^3^	Codon 219 ^4^	EEG ^5^	MRI	CSF 14-3-3	CSF RT-QuIC	Skin RT-QuIC ^1^	Skin Sampling Site
Probable sCJD ^2^										
Case 1	64	F	MM	EE	+	−	NA	NA	+	medial upper arm
Case 2	63	F	MM	EE	−	+	−	−	+	chest
Case 3	26	F	MM	EE	−	+	−	−	+	medial upper arm
Case 4	64	M	MM	EE	NA	+	−	+	+	medial upper arm
Case 5	44	F	MM	EE	−	+	+	−	+	abdomen
Case 6	59	F	MM	EE	−	+	−	+	+	medial upper arm
Case 7	72	F	MM	EE	−	+	−	−	+	abdomen
Case 8	70	M	MM	EE	−	−	+	−	+	abdomen
Case 9	69	F	NA ^6^	NA	+	+	+	−	+	inner thigh
Case 10	64	F	MM	EE	−	NA	+	+	+	medial upper arm
Case 11	68	M	MM	EE	+	+	−	+	+	posterior neck
Case 12	66	M	MM	EE	−	+	−	+	+	medial upper arm
Case 13	62	M	MV	EE	−	−	+	+	+	medial upper arm
Case 14	66	F	MM	EE	+	−	+	−	+	inner thigh
Case 15	61	M	MM	EE	+	+	NA	NA	+	medial upper arm
Case 16	70	M	MM	EE	NA	+	+	+	+	medial upper arm
Case 17	54	M	MM	EE	−	+	+	+	+	medial upper arm
Case 18	57	M	MM	EE	NA	+	+	−	+	medial upper arm
Case 19	63	M	MM	EE	−	−	+	−	+	medial upper arm
Case 20	47	F	MM	EE	−	+	−	+	+	medial upper arm
Case 21	63	M	NA	NA	−	+	−	+	+	abdomen
Case 22	49	F	MM	EE	−	+	−	−	+	medial upper arm
Case 23	54	F	MM	EE	+	+	+	−	+	medial upper arm
Case 24	69	F	MM	EE	−	+	−	−	+	posterior neck
									+	lateral malleolus
Case 25	55	F	MM	EE	−	+	+	+	+	inner thigh
									+	behind ear
Case 26	84	M	MM	EE	−	+	+	−	+	medial upper arm
									+	posterior neck
Case 27	52	M	MM	EE	−	−	+	−	−	posterior neck
									−	lateral malleolus
Case 28	68	F	MM	EE	+	−	−	+	+	posterior neck
									+	medial upper arm
Case 29	60	F	MM	EE	+	+	+	−	−	behind ear
									−	inner thigh
Case 30	67	F	MM	EE	−	−	+	−	+	medial upper arm
									+	abdomen
Case 31	53	M	MM	EE	−	+	NA	NA	−	posterior neck
									−	lateral malleolus
Case 32	81	F	MM	EE	−	−	+	−	+	inner thigh left
									+	inner thigh right
Case 33	59	F	MM	EE	−	+	+	+	+	lateral malleolus
									+	posterior neck
Case 34	62	F	MM	EE	−	+	−	+	−	posterior neck
									+	lateral malleolus
Genetic PrD										
T188K gCJD	60	F	MM	EE	+	+	−	+	+	behind ear
G114V gCJD	38	M	MV	EE	−	+	+	−	−	behind ear
D178N FFI	66	F	MM	EE	−	−	−	−	−	medial upper arm
Non-CJD										
Case 1	43	F	MM	EE	−	+	−	−	−	abdomen
Case 2	52	M	MM	EE	−	+	−	−	−	behind ear
Case 3	65	M	MM	EE	−	−	−	−	−	behind ear
Case 4	68	M	MM	EK	NA	NA	−	−	−	medial upper arm
Case 5	65	M	MM	EK	−	−	−	−	−	behind ear
Case 6	53	F	MM	EE	−	−	+	−	−	medial upper arm
Case 7	83	F	NA	NA	−	NA	+	−	−	medial upper arm
Case 8	57	F	MM	EE	−	+	+	−	−	inner thigh
Case 9	69	M	MM	EE	−	+	−	−	−	medial upper arm
Case 10	69	M	MM	EE	−	+	+	−	−	abdomen
Case 11	64	M	MM	EE	−	−	+	−	−	medial upper arm
Case 12	56	M	MM	EE	−	+	−	−	+	posterior neck
									+	lateral malleolus
Case 13	80	F	MM	EE	−	+	−	−	−	medial upper arm
									−	abdomen
Case 14	55	F	MM	EE	−	+	−	−	+	medial upper arm left
									−	medial upper arm right

^1^ The results of skin RT-QuIC was based on the final results combined with different dilutions. ^2^ sporadic Creutzfeldt-Jakob disease. ^3^ Methionine (M); Valine (V). ^4^ Glutamicacid (E); Lysine (K). ^5^ Positive (+); Negative (−). ^6^ Not Available.

**Table 3:** The RT-QuIC reactivity per well of the skin samples at the different dilutions from the patients with various diseases.

Diagnosis	Skin Sampling Site	Dilution ^2^			Final Result
		10^−2^	10^−3^	10^−4^	
Probable sCJD ^1^					
Case 1	medial upper arm	+/+/+/+	−/−/−/−	−/−/−/−	+
Case 2	chest	+/+/+/+	−/−/−/−	−/−/−/−	+
Case 3	medial upper arm	+/+/+/+	+/+/−/−/	−/−/−/−	+
Case 4	medial upper arm	+/+/+/−	−/−/−/−	−/−/−/−	+
Case 5	abdomen	+/+/+/+	+/+/+/+	−/−/−/−	+
Case 6	medial upper arm	+/+/−/−	+/−/−/−/	+/+/+/+	+
Case 7	abdomen	−/−/+	−/+/+	−/−/+	+
Case 8	abdomen	+/+/+	−/+/+	−/−/−	+
Case 9	inner thigh	−/−/−	−/−/−	+/+/+	+
Case 10	medial upper arm	+/+/+	−/−/−	−/−/−	+
Case 11	posterior neck	+/+/+	+/−/−	−/−/−	+
Case 12	medial upper arm	+/+/+	+/−/−	−/−/−	+
Case 13	medial upper arm	+/+/+/+	+/+/+/+	−/−/−/−	+
Case 14	inner thigh	+/+/+/+	+/+/+/+	+/−/−/−	+
Case 15	medial upper arm	+/+/+/+	+/+/−/−/	−/−/−/−	+
Case 16	medial upper arm	+/+/+/+	+/+/+/−	+/−/−/−	+
Case 17	medial upper arm	+/+/+/+	+/+/−/−/	−/−/−/−	+
Case 18	medial upper arm	+/+/+/+	+/+/+/+	+/+/+/−	+
Case 19	medial upper arm	+/+/+/+	+/+/+/−	+/+/−/−/	+
Case 20	medial upper arm	+/+/+/+	+/+/+/+	+/+/+/+	+
Case 21	abdomen	+/+/+/+	+/+/+/+	+/+/+/+	+
Case 22	medial upper arm	+/+/+/−	−/−/−/−	−/−/−/−	+
Case 23	medial upper arm	+/+/+/+	+/+/+/+	+/+/+/−	+
Case 24	posterior neck	+/+/+/+	+/+/+/−	+/+/+/−	+
	lateral malleolus	+/+/+/+	−/−/−/−	−/−/−/−	+
Case 25	inner thigh	+/+/+/+	+/+/−/−/	−/−/−/−	+
	behind ear	+/+/+/−	+/−/−/−/	−/−/−/−	+
Case 26	medial upper arm	+/+/+/+	+/+/+/−	−/−/−/−	+
	posterior neck	+/+/+/+	+/+/−/−/	+/−/−/−/	+
Case 28	posterior neck	+/+/+/+	+/−/−/−/	−/−/−/−	+
	medial upper arm	+/+/+/−	−/−/−/−	−/−/−/−	+
Case 30	medial upper arm	+/+/+/+	+/+/+/+	+/−/−/−/	+
	abdomen	+/+/+/+	+/+/+/−	−/−/−/−	+
Case 32	inner thigh left	+/+/+/+	+/+/+/+	+/+/+/−	+
	inner thigh right	+/+/+/+	+/+/+/+	+/+/−/−	+
Case 33	lateral malleolus	+/+/+/+	+/+/−/−/	−/−/−/−	+
	posterior neck	+/+/+/+	+/+/+/+	+/+/−/−/	+
Case 34	posterior neck	+/−/−/−/	−/−/−/−	−/−/−/−	−
	lateral malleolus	+/+/+/+	+/+/−/−/	+/−/−/−/	+
gPrD					
T188K gCJD	behind ear	+/+/+/+	+/+/+/+	−/−/−/−	+
Non-CJD					
Case 12	posterior neck	+/−/−/−/	+/+/+/+	−/−/−/−	+
	lateral malleolus	+/+/+/−	−/−/−/−	−/−/−/−	+
Case 14	medial upper arm left	+/+/+/−	+/+/−/−	−/−/−/−	+
	medial upper arm right	+/−/−/−	−/−/−/−	−/−/−/−	−
Total (pos/total, %)	/	39/44 (88.6%)	28/44 (63.6%)	11/44 (25.0%)	/

^1^ sporadic Creutzfeldt-Jakob disease. ^2^ Positive (+); Negative (−).

**Table 4:** Comparisons of clinical and laboratory data between skin RT-QuIC positive and negative groups.

	Positive	Negative	*p*-Value ^1^
Age (median, min-max) (y ^2^)	61.5 (26–84)	60.9 (38–83)	0.86
Gender (M/F ^3^)	13/21	10/7	0.17
Codon 129 (MM/MV ^4^)	31/1	15/1	0.62
Codon 219 (EE/EK ^5^)	32/0	14/2	0.16
EEG (+/− ^6^)	8/23	1/15	0.06
MRI (+/−)	25/8	9/6	0.28
CSF 14-3-3 (+/−)	15/17	7/8	0.99

^1^ Levene’s test and Student’s *t*-test were used. *p* ≤ 0.05 was considered to be statistically significant. ^2^ years (y). ^3^ Female (F); Male (M). ^4^ Methionine (M); Valine (V). ^5^ Glutamicacid (E); Lysine (K). ^6^ Positive (+); Negative (−).

## Data Availability

All data is available upon request.
